# Characterization and Genomic Analysis of the First Podophage Infecting *Shewanella*, Representing a Novel Viral Cluster

**DOI:** 10.3389/fmicb.2022.853973

**Published:** 2022-04-01

**Authors:** Yue Dong, Kaiyang Zheng, Xiao Zou, Yantao Liang, Yundan Liu, Xiang Li, Hongbing Shao, Yeong Yik Sung, Wen Jye Mok, Li Lian Wong, Andrew McMinn, Min Wang

**Affiliations:** ^1^College of Marine Life Sciences, Institute of Evolution and Marine Biodiversity, Frontiers Science Center for Deep Ocean Multispheres and Earth System, Key Lab of Polar Oceanography and Global Ocean Change, Ocean University of China, Qingdao, China; ^2^Qingdao Central Hospital, Qingdao, China; ^3^UMT-OUC Joint Center for Marine Studies, Qingdao, China; ^4^Institute of Marine Biotechnology, Universiti Malaysia Terengganu (UMT), Kuala Nerus, Malaysia; ^5^Institute for Marine and Antarctic Studies, University of Tasmania, Hobart, TAS, Australia; ^6^The Affiliated Hospital of Qingdao University, Qingdao, China

**Keywords:** *Shewanella*, podophage, genomic analysis, phylogenetic analysis, novel viral cluster

## Abstract

*Shewanella* is a common bacterial genus in marine sediments and deep seas, with a variety of metabolic abilities, suggesting its important roles in the marine biogeochemical cycles. In this study, a novel lytic Shewanella phage, vB_SInP-X14, was isolated from the surface coastal waters of Qingdao, China. The vB_SInP-X14 contains a linear, double-strand 36,396-bp with the G + C content of 44.1% and harbors 40 predicted open reading frames. Morphological, growth, and genomic analysis showed that it is the first isolated podovirus infecting *Shewanella*, with a short propagation time (40 min), which might be resulted from three lytic-related genes. Phylogenetic analysis suggested that vB_SInP-X14 could represent a novel viral genus, named Bocovirus, with four isolated but not classified phages. In addition, 14 uncultured viral genomes assembled from the marine metagenomes could provide additional support to establish this novel viral genus. This study reports the first podovirus infecting *Shewanella*, establishes a new interaction system for the study of virus–host interactions, and also provides new reference genomes for the marine viral metagenomic analysis.

## Introduction

Viruses are the most abundant “life forms” in the ocean, with the abundance about 10 times greater than the microbes, while most of them are bacteriophages (Wigington et al., 2016; Gong et al., 2018). The spatial and temporal distribution patterns of marine bacteriophages are tightly coupled with their host cells, suggesting their important roles in marine microbial ecosystems. In the surface oceans, marine phages caused approximately 10 - 50% of bacterial mortality through infection and lysis (Fuhrman, 1999; Gomez-Gil et al., 2014). While in the deep seas, phage lysis could cause up to 100% of bacterial mortality due to the lack of grazing effect from the eukaryotes (Suttle, 1994; Macián et al., 2005; Bartlau et al., 2021).

Bacterial cells of the genus *Shewanella*, order Gammaproteobacteria, lives in almost all habitats on the Earth, from the tropical to the frigid zones, from the land to the sea, and from the surface ocean to the hadal trench, and even to the marine sediments (Kato and Nogi, 2001; Lemaire et al., 2020). This bacterial genus is also ubiquitous in the complex communities of aquatic and sedimentary ecosystems (Hofle and Brettar, 1996), and is one of the typical bacterial genera of the deep sea and ocean sediments (Kato and Nogi, 2001). *Shewanella* has the omnipotent electron-accepting ability, possessing different metabolic abilities and great potential in microbial fuel cells (Fredrickson et al., 2008; Yang et al., 2020). In addition, *Shewanella* has dissimilatory metal reduction ability, which is important in the treatment of heavy metal pollution (Fredrickson et al., 2008). Considering the ecological significance of *Shewanella*, the genomic and ecological study of their phages has just begun to be addressed (Lemaire et al., 2020). Currently, 23 Shewanella phages have been isolated and deposited in GenBank (November 2021). All Shewanella phages were classified into the order Caudovirales, including 10 isolates within the family Siphoviridae, 10 isolates within the family Myoviridae, and two isolates within the family Chaseviridae (Table 1). However, Shewanella phage within Podoviridae is not reported yet.

**TABLE 1:** The information of all isolated Shewanella phages.

Family	Host strain	Phage	Accession number	Genome size(kb)	GC content	Number of ORF
Podoviridae	*Shewanella inventionis*	Shewanella phage X14	MK796797	36396	44.10	40
*Chaseviridae*	*Shewanella putrefaciens*	Shewanella virus Spp001	NC_023594	54791	49.42	67
*Chaseviridae*	*Shewanella putrefaciens*	Shewanella virus SppYZU05	NC_047824	54319	50.64	69
*Siphoviridae*	*Shewanella indica*	Shewanella phage S0112	MK675901	62286	44.7	101
*Siphoviridae*	Shewanella	Vibrio phage 1.049.O._10N.286.54.B5	MG592431	45021	41.6	60
*Siphoviridae*	Shewanella	Vibrio phage 1.050.O._10N.286.48.A6	MG592432	45285	41.64	60
*Siphoviridae*	Shewanella	Vibrio phage 1.076.O._10N.286.51.B7	MG592452	47914	42.84	73
*Siphoviridae*	Shewanella	Vibrio phage 1.083.O._10N.286.52.B9	MG592458	45120	38.71	61
*Siphoviridae*	Shewanella	Vibrio phage 2.096.O._10N.286.48.B5	MG592666	44683	38.78	57
*Siphoviridae*	Shewanella sp. M16	Shewanella phage vB_SspS_KASIA	MZ568826	91102	40.23	145
*Siphoviridae*	Shewanella sp.	Shewanella sp. phage 1/44	NC_025463	49640	39.82	75
*Siphoviridae*	Shewanella sp.	Shewanella sp. phage 3/49	NC_025466	40161	41.97	70
*Siphoviridae*	Shewanella sp. M16.	Shewanella phage vB_SspS_MuM16-1	MZ568827	37883	47.83	52
*Myoviridae*	Shewanella	Shewanella sp. phage 1/40	NC_025470	139004	36.89	239
*Myoviridae*	Shewanella	Shewanella sp. phage 1/4	NC_025436	133824	36.86	238
*Myoviridae*	Shewanella	Shewanella sp. phage 1/41	NC_025458	43510	42.7	69
*Myoviridae*	Shewanella	Shewanella phage Thanatos-1	MT457552	160584	34.51	208
*Myoviridae*	Shewanella	Shewanella phage Thanatos-2	MT457553	155580	34.77	195
*Myoviridae*	Shewanella baltica	Shewanella phage SppYZU01	KY622015	43567	55.72	49
*Myoviridae*	Shewanella fidelis	Shewanella phage SFCi1	KX196154	42279	59.07	29
*Myoviridae*	Shewanella	Vibrio phage 1.081.O._10N.286.52.C2	MG592456	239318	42.41	354
*Myoviridae*	Shewanella sp. M16	Shewanella phage vB_SspM_MuM16-2	MZ568828	33702	46.85	50
*Myoviridae*	Shewanella sp. M16	Shewanella phage vB_SspM_M16-3	MZ568829	40166	46.8	63

In this study, we isolated and sequenced the first podovirus infecting *Shewanella*, named vB_SInP-X14, from the surface coastal waters of Qingdao, China. Morphological, growth, genomic, and phylogenetic characteristics of vB_SInP-X14 were revealed. We demonstrated that vB_SInP-X14 shows a high level of genome similarities to four isolated but not classified phages and 14 uncultured viral genomes (UViGs) assembled from the marine metagenomes. Hence, we proposed a novel viral cluster at the genus level, named *Bocovirus*.

## Materials and Methods

### Phage and Its Host Isolation and Purification

*Shewanella inventionis* ZQ14 and its phage vB_SInP-X14 were both isolated from the coastal waters of Qingdao, China (36.063°N, 120.320°E). Diluted 1 ml seawater to 10^–5^ step by step and took 200 μL diluted seawater, inoculated it on 2216E solid medium by the plate coating method, and cultured it in an incubator at 28°C and 120 rpm. *Shewanella inventionis* ZQ14 was purified more than three times in the 2216E medium. Then, the 16s rRNA of the purified *Shewanella inventionis* ZQ14 was amplified by PCR and sequenced. After searching by BLASTn, the 16SrRNA similarity between the *Shewanella inventionis* ZQ14 and *Shewanella inventionis* KX27 (T) was 99.69%.

Phage was isolated by the double-layer plate method (Zhang W. et al., 2021). The same seawater sample was filtered through the 0.22 μm pore-size membranes (Millipore) to obtain viral water. The 200 μl host bacterial (*Shewanella inventionis* ZQ14) culture in the logarithmic growth stage was inoculated with 200 μl filtered seawater and incubated for 15 min. A single plaque was collected and placed into SM buffer (50 mM Tris-HCl, 0.1 M NaCl, and 8 mM MgSO4 [pH 7.5]) at 4°C. The viral mixture was passed through a 0.22-μm membrane filter and allowed to infect the host again. This step was repeated five times to obtain purified viral stock.

The purified virus was amplified to 500 ml using the reinfection of *Shewanella inventionis* ZQ14. Then viral culture was passed through a 0.22-μm membrane filter (Millipore) to remove the bacteria. The virus culture was concentrated to 3 ml with an ultrafiltration tube (Millipore Amicon Ultra-15) at 5,000 g (Zhang W. et al., 2021).

### Transmission Electron Microscopy

The vB_SInP-X14 morphology was examined by transmission electron microscopy (TEM). Briefly, 20 μl purified and concentrated phage solution was negatively stained with phosphotungstic acid (1%w/v, pH 7.0) for 5 min (Haq et al., 2012). The electron micrographs of vB_SInP-X14 were captured by JEOL Model JEM-1200EX TEM at 100 kV.

### One-Step Growth, Thermal and pH Stability Assays

The 1 ml bacterial solution and 1 ml purified viral solution were incubated at 26°C for 15 min with MOI 0.1, the non-absorbed virus was removed by centrifugation, and the precipitate was washed three times with 1 ml fresh 2216E liquid to remove the remaining unabsorbed phage. Sampling was done every 5 min in the first hour and every 10 min from the second hour. Viral abundance was determined by the method of double-layer agar plaque assay. The number of plaques in different periods was counted to draw the growth curve. Three parallel experiments were conducted for this assay (Cai et al., 2019).

Liquid media of 2216E were prepared with pH of 3, 4, 5, 6, 7, 8, 9, 10, 11, and 12 and sterilized at 121°C for 20 min (Liu et al., 2019, 2021). The 100 μl phage solution (initial titer ∼ 10^8^ PFU/ml) was diluted with 1 ml medium of different pH and placed at room temperature for 2 h. The 200 μl phage solution treated with different pH was mixed with 200 μl of the host bacterial solution and the host was in the logarithmic growth stage, whose concentration was 2 × 10^7^ cfu/ml. After infection for 15 min, the double-layer plate (solid 2216E medium: agar 15 wt.%, semi-solid 2216E medium 7.5 wt.%, agar brand: Solarbio, Beijing) was poured and cultured at a constant temperature overnight at 26°C. Then the number of plaques was counted to draw the phage survival curve at different pH, and carried out three parallel experiments.

Several copies of the same phage solution (titer ∼ 10^8^PFU/mL) were treated at - 20°C, 4°C, 25°C, 35°C, 45°C, 55°C, 65°C, and 75°C, respectively, for 2 h (Zhang et al., 2020). The host bacterial solution in the logarithmic growth stage was vortex mixed with the treated phage solution. After infection for 15 min, the double-layer plate was poured and cultured at a constant temperature overnight. The plaques on the plate were counted and the phage growth curve related to temperature was drawn. We carried out three parallel experiments.

### Phage DNA Extraction, Sequencing, and Annotation

The DNA extraction of vB_SInP-X14 was performed by Virus DNA Kit (OMEGA).

The purified phage genomic DNA was sequenced by Novogene Company (Tianjin, China), using Illumina Miseq 2 × 300 paired-end method. The sequencing library used NEBNext Ultra DNA Library Prep Kit for Illumina (NEB, United States). In short, the 1 μg DNA sample was cut to 350 bp size for Illumina sequencing and further PCR amplification. To ensure the accuracy and reliability of subsequent information analysis results, the raw data was filtered to obtain effective data (clean data), and clean data produced 4,073,908 reads. Clean data were assembled using the software ABySS (v1.3.7) (Simpson et al., 2009) with multiple-Kmer parameters. The GapClose (v1.12) software was applied to fill the gap of preliminary assembly results with default parameters. The fragments shorter than 500 bp were filtered and the final results were obtained. BWA (bwa-0.7.8) software was used to compare the reads to the assembled genome sequence, and SAMTOOLS (v 0.1.19) software was used to interpret and count the coverage of the reads to the assembled genome sequence. Both BWA and SAMTOOLS software used default parameters. The average coverage of reads on the genome was 33,330. Orientation was obtained by using the program BreakDancer (Fan et al., 2014). The genome sequence Shewanella phage vB_SInP-X14 has been deposited in GenBank, whose accession number is MK796797. The cumulative GC skew^1^ analysis was used to detect the origin and terminus of replication of the phage genome (Uchiyama et al., 2008; Zhang W. et al., 2021). The prediction of the putative open reading frames (ORFs) was combined with several different software, including prodigal (Hyatt et al., 2010), GeneMarkS sever (Besemer et al., 2001), and RAST (Brettin et al., 2015). All ORFs were annotated by Pfam with default parameters (Mistry et al., 2021), BLASTp (*E*-value < 1e-5) against by the Non-Redundant (nr) protein database of the GenBank, HHpred simultaneously based on four databases (PDB_mmCIF70_13_Jul, Pfam-A_v34, COG_KOG_v1.0, NCBI_Conserverd_Domains (CD)_v3.18) (Söding et al., 2005). The genome mapping was visualized by CLC Main Workbench 20. The tRNAscan-SE program predicted tRNA sequence (Lowe and Chan, 2016).

### Search for Viral Sequences Homologous to VB_SInP-X14 in NCBI and IMG/VR

To expand the homologous sequences of vB_SInP-X14, genome sequence of vB_SInP-X14 was searched as a query against the NCBI database, searching through BLASTn (E-value < 1e -10, Query Cover > 25%), and against the Integrated Microbial Genomes/Virus (IMG/VR) through Diamond BLASTp (v.0.9.21) (*E*-value < 1e-10, Query Cover > 50%). Sequences shared more than 50% ORFs of vB_SInP-X14 were considered as candidate sequences (Buchfink et al., 2015).

### Network Analysis

A total of 65,899 Caudovirales phages were downloaded from NCBI-virus. The pre-experiment of protein sharing between phages was carried out by using vConTACT to find phages linked to vB_SInP-X14. Then, according to the pre-experiment results, only added vB_SInP-X14-related phages and NCBI-RefSeq for cluster analysis to facilitate the data visualization. Accordingly, 4,406 reference genomic sequences of isolated Caudovirales, were selected and combined with the above homologous sequences to vB_SInP-X14 in the IMG/VR dataset. All proteins were compared using all-*versus*-all DIAMOND BLASTp (E-value ≤ 1E-10, coverage ≥ 50%, amino acid identity ≥ 30%). Markov clustering algorithm (MCL) was used to divide protein clusters (PCs) (Enright et al., 2002). Then vConTACT 2.0 was used to calculate the similarity score between genomes. All predicted proteins were combined as a database with DIAMOND BLASTp; therefore, the database in vConTACT chose “none.” Viral clusters were defined using ClusterONE (Nepusz et al., 2012). To show the detailed cluster analysis result of vB_SInP-X14, the results of vConTACT analysis were screened. Only the classified phage genomes of Podoviridae in the International Committee on Taxonomy of Viruses (ICTV) were selected and combined with homologous sequences to vB_SInP-X14 as the source. The phages that generated weight with the source were selected and displayed in the detailed figure. The network diagram was visualized by Gephi.

### Phylogenetic Analyses

All 23 isolated Shewanella phages in GenBank (Table 1) were used to build a whole-genome tree with Viptree^2^ (version 2.0) and analyze their similarities. The Viptree calculates the sequence similarities of the whole genomes according to tblastx, and generates the “proteome tree” of the viral genome sequences (Nishimura et al., 2017). In addition, a separate phylogenetic analysis of vB_SInP-X14 was constructed using Viptree.

A phylogenetic tree based on the whole genome, including at least one member of all genera within Podoviridae family in the ICTV, was constructed. The whole-genome phylogenetic tree including 82 viral isolates was constructed by the Virus Classification and Tree Building Online Resource (VICTOR) (Meier-Kolthoff and Göker, 2017) based on nucleotide, and the treefile of GBDP_Trimming_D6_FASTME was chosen and visualized by iTol (V.5) (Letunic and Bork, 2019). The whole-genome Average Nucleotide Identity (ANI) was computed by fastANI (Jain et al., 2018), then visualized by pheatmap in R. In addition, this result also verifies the results of VICTOR and fastANI. According to the results of the phylogenetic tree and ANI analysis, some viral isolates and representative UViGs similar to vB_SInP-X14 were selected to perform the whole genome gene comparison map with Viptree, and the protein clustering through All-to-all BLASTp analysis was performed by Orthofinder (version: 2.5.2) using the diamond blastp flag of *E*-value < 1e-5, Query Cover > 50%, and identity > 30% (Emms and Kelly, 2015).

### Ecological Distribution in the GOV2.0

The relative abundances of vB_SInP-X14 were expressed as tpm (transcripts per million) and calculated by CoverM (v0.3.1) (parameters: -m tpm –min-read-percent-identity 0.95 –min-read-aligned-percent 0.75) (Li B. et al., 2010; Li et al., 2021). The quality-controlled reads from the 154 Global Ocean Viromes (GOV 2.0) dataset were mapped to the vB_SInP-X14 contig by minimap2 (v2.17) program. Several other phages were selected as references, including the representative pelagibacter phages (HTVC010P, HTVC019P, HTVC011P), cyanophages (P-SSB7, P-SSM7) (Labrie et al., 2013), Puniceispirillum (SAR116) phage HMO-2011 (Zhao et al., 2013), Roseophage SIO1 (Angly et al., 2009), two phages similar to vB_SInP-X14 (Vibrio phage VvAW1 and Thalassomonas phage BA3) (Efrony et al., 2009; Nigro et al., 2012), and other Shewanella phages. Five marine viral ecological zones (VEZs) defined by the Global Ocean Viromes (GOV 2.0) dataset are Arctic (ARC), Antarctic (ANT), temperate and tropical epipelagic (EPI), temperate and tropical mesopelagic (MES), and bathypelagic (BATHY) (Gregory et al., 2019). The relative abundance of viruses in the five VEZs were added and transformed into log_10_, and the subsequent results were visualized by heatmap and histogram.

## Results and Discussion

### Morphology and Biological Characterization of vB_SInP-X14

**FIGURE 1:**
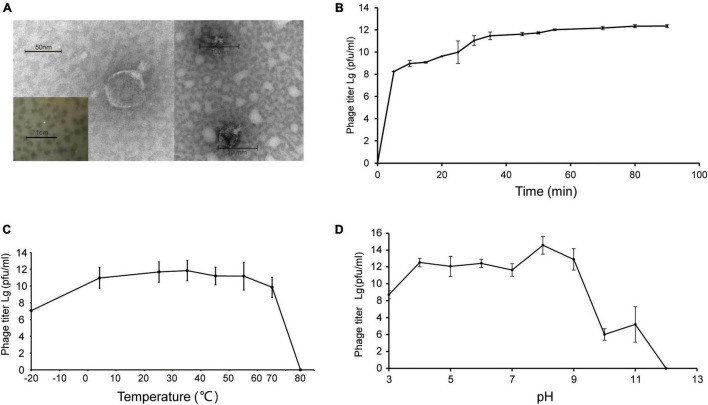
**(A)** Transmission electron micrograph of Shewanella phage vB_SInP-X14. Scale bar, 50 nm. And the plaques of the Shewanella phage vB_SInP-X14 on the lawn of *Shewanella inventionis* ZQ14. Scale bar is 1 cm. **(B)** One-step growth curve of Shewanella phage vB_SInP-X14, each point is the average of three independent experiments. The curve of thermal stability **(C)** and pH stability **(D)** of Shewanella phage vB_SInP-X14.

Shewanella phage vB_SInP-X14 was isolated from surface coastal waters of Qingdao, China (36.063°N, 120.320°E), regarding *Shewanella inventionis* ZQ14 as the host. vB_SInP-X14 formed clear plaques with 2 mm diameter in double-layer agar (Figure 1A). The typical podoviral morphology of vB_SInP-X14 was observed by TEM, which had an icosahedron head (∼59 nm in diameter on average) and a short non-contractible tail (∼24 nm long on average) (Figure 1A).

Experiments of viral one-step growth, pH stability, and temperature tolerance were performed to characterize the biological features of vB_SInP-X14. From the result of viral one-step growth analysis (Figure 1B), the latent period is less than 5 min and the rise period is approximately 40 min. The short latent period of vB_SInP-X14 suggested that the viruses could be rapidly absorbed and injected into cells, and indicated the unique cellular virulence of vB_SInP-X14 (Ma et al., 2021). The growth curve of this trend has also been reported (Chan et al., 2014; Li et al., 2016). In the temperature stability experiment, the phage showed the maximum titer at 35°C. The titer of vB_SInP-X14 was at a high level when the temperature ranged from −20°C to 65°C (Figure 1C); however, the phage titer decreased linearly at more than 65°C, and completely lost its activity at 75°C. The optimum pH range of vB_SInP-X14 is between four and nine (Figure 1D). When the pH exceeds nine, the phage titer decreases sharply and until the pH is 12, the phage loses its activity.

### The Genomic Characteristic and Annotation

**FIGURE 2:**
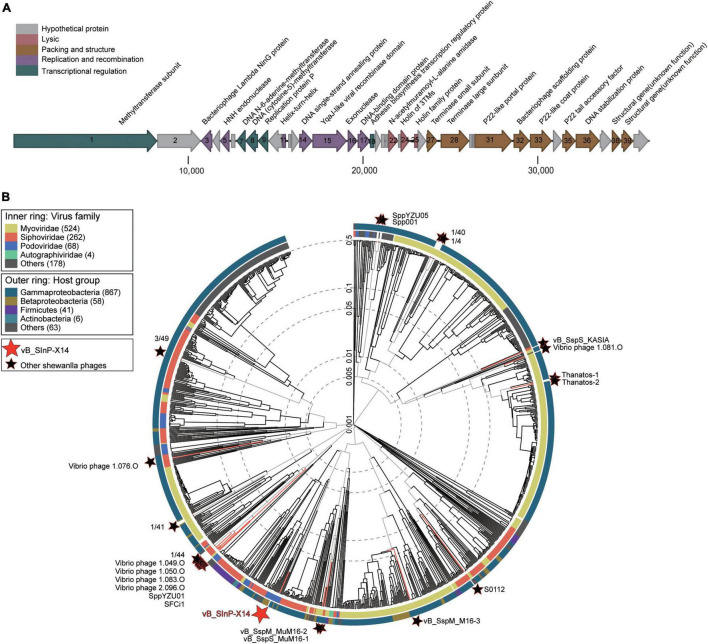
Genome and phylogenetic analysis of Shewanella phage vB_SInP-X14 with isolated phage genomes. **(A)** Genome map and functional annotation of the predicted proteins of Shewanella phage vB_SInP-X14. The length of each arrow represents each gene length. The functional genes are divided into five parts with different colors. **(B)** Phylogenetic tree of all isolated Shewanella phages by VipTree. The red star is Shewanella phage vB_SInP-X14, black stars are other Shewanella phages, and the others were the 100 most similar to each Shewanella phage selected.

Phage vB_SInP-X14 encapsulated a double-strand DNA genome with a length of 36,396 bp, G + C content of 44.1%. The result of the cumulative GC Skew analysis indicated that the origin and terminus of replication were at the regions 200 nt with the lowest value and 36,300 nt with the highest value, respectively (Supplementary Figure 3). A total of 40 ORFs were predicted, and 24 of which have orthologs with known functional genes in the public databases (Supplementary Table 1). The genome of vB_SInP-X14 is modularized by divergent functions and formed five gene clusters in the genomes. The replication/recombination and transcriptional regulation modules were viral early gene clusters, containing six (ORFs 3,5,11, and 14-17) and five (ORFs 1, 7-9, and 18) ORFs, respectively. The structural and packaging module, lytic module were viral late gene clusters, containing nine (ORFs 27, 28, 31, 32, 33, 35,36, 38, and 39) and three (ORFs 22, 24, and 25) ORFs, respectively (Figure 2A). Transcriptional regulation and replication/recombination modules accounted for large proportions in the genome of vB_SInP-X14. P22-homologous structural and packaging-related proteins were detected in the genome of vB_SInP-X14. Two P22-like packaging genes were annotated, including the P22 tail accessory factor (ORF 35) and the P22-like portal protein (ORF 31). In addition, vB_SInP-X14 contains a P22-like capsid protein (ORF 33).

In the structural and packaging module, ORFs 31, 32, and 33 (locus-tags: X14_000031, X14_000032, and X14_000033) encoded portal protein, scaffolding protein, and coat protein, respectively, which are necessary for procapsid formation of tailed phages. The bacteriophage scaffold protein (ORF32) is an essential protein for the formation of a procapsid (Black, 1995). The intermediate structure of the procapsid is formed by the assembly of scaffold protein and viral coat protein, in which the scaffold protein is located in the coat protein subunit during the arrangement of the icosahedron. In subsequent steps, the scaffold protein molecules are released, leaving shell proteins that cannot be self-assembled (Sun et al., 2000). Then the viral genome is packaged into a preformed procapsid. In the DNA phage, it is composed of two packaging proteins, named terminase large subunit and terminase small subunit (TerL and TerS) (Casjens, 2011). They are assembled into the dodecamer of portal protein and insert DNA into the empty capsid with high precision and efficiency, and the whole process needs to be driven by ATP (Roy and Cingolani, 2012). In the TerL, two or possibly three ATP-binding sites are expected, and the TerL usually shows a specific affinity for portal protein. In contrast, the TerS seems to regulate the activity of the large subunit (Rao and Black, 1988; Black, 1995). Besides, similar to many other phages, the TerS and TerL subunits are adjacent, which are ORFs 27 and 28 in the genome of vB_SInP-X14, respectively (Duhaime et al., 2011).

Three lytic-associated ORFs were encoded by the virus genome, suggesting that the life strategy of vB_SInP-X14 might be lytic, which was in accordance with the results of the one-step growth curve The lytic module includes two holin proteins (ORFs 24 and 25) and one endolysin *N*-acetylmuramoyl-L-alanine amidase (ORF 22, locus-tag: X14_000022). ORFs 24 and 25 belong to the holin family, and their potential functions appear to be the transport of murein hydrolases across the cytoplasmic membrane to the cell wall where these enzymes hydrolyze the cell wall polymer as a prelude to cell lysis (Schmidt et al., 1996). The potential function of ORF 22 is to dissolve the amide bond between *N*-acetylmuramoyl and L-amino acids in bacterial cell walls (Hynes et al., 2012).

Apparent modularization was observed for the genome of vB_SInP-X14 on the basis of the ORFs arrangement (Figure 2A).

### Phage VB_SInP-X14 Represents a Novel Viral Cluster

Phage vB_SInP-X14 is the first isolated podovirus infecting *Shewanella*, distinct from other Shewanella phages. To identify the taxonomic position of the phage vB_SInP-X14, the phylogenetic tree based on the viral proteome of whole genomes was established by Viptree, including all isolated Shewanella phages (Table 1) and 100 viruses closely related to Shewanella phages (Wang et al., 2021). The result showed that vB_SInP-X14 was far away from other Shewanella phages (Figure 2B). Therefore, vB_SInP-X14 can be clustered with other podovirus within Podoviridae family (Figure 2B and Supplementary Figure 2). The hosts of these podoviruses are Betaproteobacteria and Gammaproteobacteria, while *Shewanella* is within the Gammaproteobacteria class.

**FIGURE 3:**
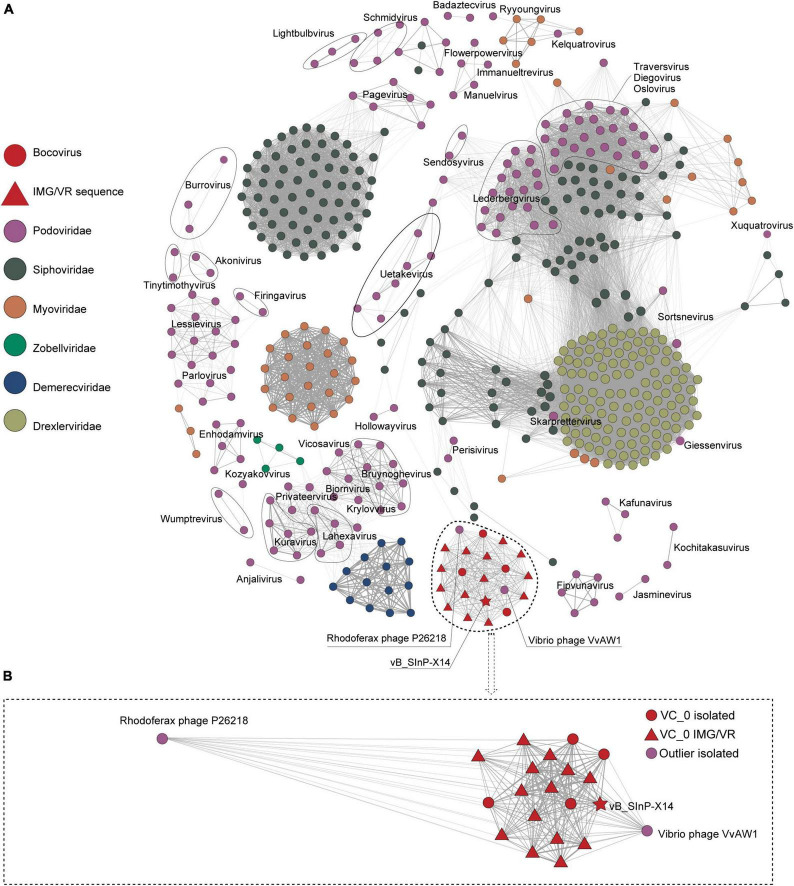
Gene content–based viral network of Shewanella phage vB_SInP-X14, *Caudovirales* virus from NCBI-Ref database, and related UViGs from IMG/VR dataset. To highlight the key result, we only showed the nodes associated with podovirus (classified in ICTV) and removed the singleton nodes. The network was visualized using Gephi version 0.9.2. **(A)** The Fruchterman Reingold layout (Gephi) explores the clustering status of the groups represented by vB_SInP-X14 globally. **(B)** Using the force atlas layout, the local network diagram in which the nodes have a direct linkage to vB_SInP-X14 is shown. The edges represent the similarity scores between genomes based on shared gene content.

Phage vB_SInP-X14 was also different from other isolated phages in the order Caudovirales. After searching against the entire NCBI database, we found four homologous phages to vB_SInP-X14, which were unclassified at the genus level and not included in the NCBI-ref database. And the genomes of three phages were incomplete. Through searching against the IMG/VR database (Zhan and Chen, 2019), we detected 14 non-redundant homologous UViGs closely related to vB_SInP-X14 (Supplementary Table 2). In the network diagram of the pre-experiment, only phages related to vB_SInP-X14 were presented (Supplementary Figure 1). The network diagram (Figure 3), vB_SInP-X14 has linkages between six isolated phages. Among them, vB_SInP-X14, four isolated phages without genus-level taxonomy information (Thalassomonas phage BA3, Vibrio phage 1.183.O, Vibrio phage 1.184.A, Vibrio phage 1.211.A), and 14 UViGs from IMG/VR were classified into the same viral cluster (VC 0_0). Although Vibrio phage VvAW1 and Rhodoferax phage P26218 have linkage with vB_SInP-X14, they were not classified under VC 0_0 and used as outlier references (Figure 3B). According to these results, we suggest that the members of VC 0_0 could represent a new genus, named here as *Bocovirus*. In addition, the VC 0_0 is a relatively independent cluster away from other defined viral genera (Figure 3A).

**FIGURE 4:**
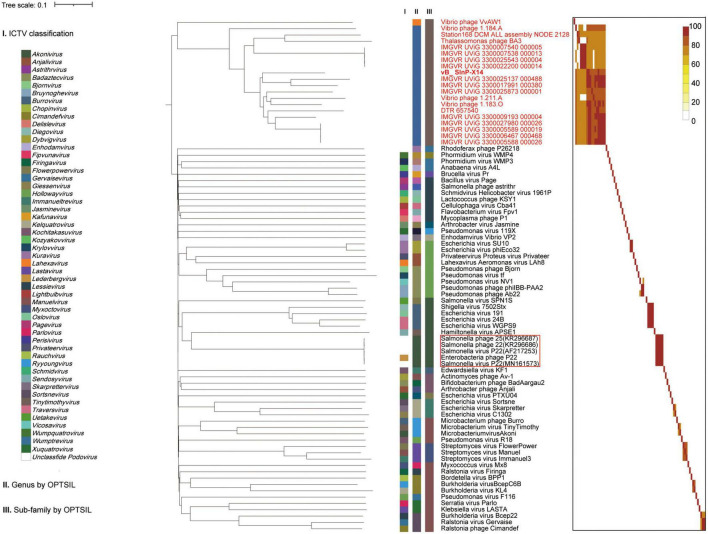
Whole-genome based phylogenic tree and gene content–based viral network of Shewanella phage vB_SInP-X14, *Caudovirales* virus from NCBI database, and related uncultured viral genome sequences (UViGs) from Integrated Microbial Genomes/Virus (IMG/VR). Whole-genome–based phylogenic tree constructed by Virus Classification and Tree Building Online Resource (VICTOR) with the formula d6 and average nucleotide identity (ANI) grouping. The phylogenic tree consists of 82 phage genomes, including 56 *Podoviridae* strains classified by ICTV, 1–2 representative strains selected from each genus; 14 UViGs in IMG/VR and five isolated but unclassified strains, which are similar to Shewanella phage vB_SInP-X14. Shewanella phage vB_SInP-X14 and its similar sequences (names in red) were classified into the same virus cluster. Three series of color boxes behind the tree indicate: I. Genera classified by ICTV; II. Genus-leveled VCs of all these phages classified by OPTSIL; III. Sub-family-leveled viral clusters of all these phages classified by OPTSIL.

The phylogenetic tree established by VICTOR is an effective tool for viral classification. vB_SInP-X14 and its homologous sequences are novel in the VICTOR phylogenetic tree, after comparing with all the classified Podoviridae phages in the ICTV. The unclassified branch represented by vB_SInP-X14 is distant from other classified viruses (Figure 4). According to the whole-genome phylogeny and OPTSIL classification, 20 phages and UViGs represented by vB_SInP-X14 can be divided into the same subfamily, while 19 of them except Vibrio phage VvAW1 can be grouped into a novel genus (Zhang Z. et al., 2021). In addition, The FastANI heatmap showed that the ANI values of the 19 phages and UViGs ranged from 76.39 to 91.26% (Figure 4), which is higher than the threshold ANI values (> 70%) for the classification of the same genus by the ICTV (Turner et al., 2021).

Virus classification is a process of continuous improvement. At present, there is more and more software to help us classify viruses. Therefore, the combination usage of a variety of methods is necessary to achieve a persuasive result. This study used the method of Turner et al. (2021), combined the results at both nucleic and amino acid levels, and used multiple analysis methods to classify and analyze the vB_SInP-X14.

### Comparative Genomic Analysis Between VB_SInP-X14 and Closely Related Phages and UViGs

**FIGURE 5:**
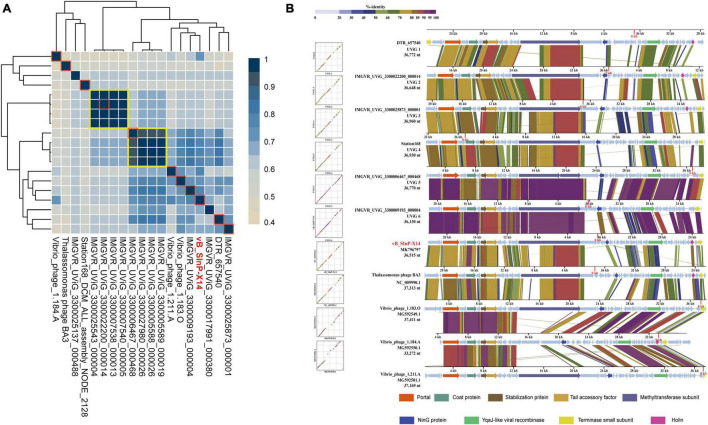
Relationship between Shewanella phage vB_SInP-X14 and similar phage genomes at the protein level. **(A)** Protein level heatmap clustering of the 19 vB_SInP-X14 and homologous genomes, the gene sharing rate among 19 genomes were calculated by all-to-all BLASTp at E value < 1e-5, identity > 30%, Qcover > 50%. **(B)** Comparative genomic analysis of 11 sequences (frame in red) selected from the protein level heatmap clustering. One representative sequence was selected from each of the two big groups at the protein-level heatmap clustering (shown in yellow boxes). Two incomplete genomes were discarded. The functional genes shared between them were marked in different colors.

Exploring homologous genes between different genomes is a common research method for phylogenetic analysis (Brüssow and Hendrix, 2002; Hatfull and Hendrix, 2011). The 19 phages and UViGs represented by vB_SInP-X14 in the whole-genome phylogenetic tree were selected for the detailed genomic and comparative genomic analysis (Figure 5). The protein homology heatmap using all-*versus*-all BLASTp showed that their gene sharing rates were higher than 40% (Figure 5A). The gene sharing rate of vB_SInP-X14 with other genomes ranged from 54 to 76%. In the protein homology heatmap, two groups have a high gene sharing rate and selected one sequence from each of these two groups (Figure 5A). According to the above results, two incomplete genome sequences with shorter lengths were discarded, and the rest 11 genome sequences were selected for the comparative genomic analysis. The result showed that conserved genes are distributed in almost every module, except lytic (Figure 5B). In the packaging module, including terminase small subunit, portal protein, tail accessory factor, and stabilization protein are conservative in the 11 genome sequences. In structural genes, the 11 genome sequences shared a conserved coat protein. The methyltransferase subunit in the transcriptional regulation module, that is, the domain contained by the largest gene in the whole genome, except Vibrio_ phage_ 1.184. A, all 10 genomes are conserved. In addition, only one lytic-related gene was detected in several sequences, and there were no conserved cleavage genes in these 11 genome sequences, which also shows the particularity of vB_SInP-X14, suggesting their short lytic life cycle (Figure 1B).

### Ecological Distribution of VB_SInP-X14 in the Ocean

**FIGURE 6:**
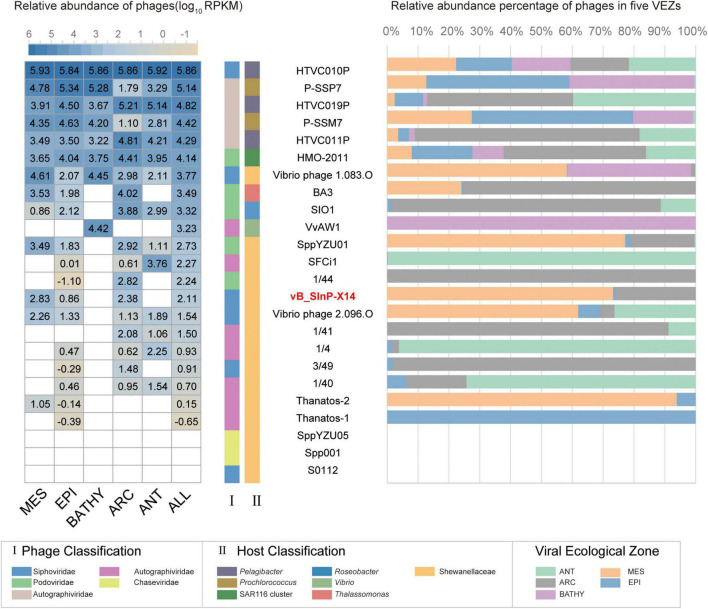
The relative abundance of Shewanella phage vB_ SInP-X14 in 154 viromes of the Global Ocean Viromes (GOV 2.0) data set. The relative abundance was expressed as tpm (transcripts per million mapped reads) and values calculated by CoverM (v0.3.1). Values were normalized by the number of databases of each viral ecological zone (VEZ) for the right bar chart, and the result was log10 transformed for the left heatmap. Representative pelagibacter phages, cyanophages, Puniceispirillum (SAR116) phage HMO-2011, two phages similar to vB_SInP-X14 in the genome (Vibrio phage VvAW1 and Thalassomonas phage BA3), and other Shewanella phages were added as references. Five marine VEZs are Arctic (ARC), Antarctic (ANT), temperate and tropical epipelagic (EPI), temperate and tropical mesopelagic (MES), and bathypelagic (BATHY).

The biogeographical distribution of vB_SInP-X14 was characterized in the 154 viral metagenomes from five VEZs of the Global Ocean Viromes (GOV2.0) dataset: Arctic (ARC), Antarctic (ANT), temperate and tropical epipelagic (EPI), temperate and tropical mesopelagic (MES), and bathypelagic (BATHY) (Gregory et al., 2019). The results confirmed that HTVC010P is one of the most abundant viruses in the ocean (Figure 6; Zhao et al., 2013). In addition, Prochlorococcus phage P-SSP7 and P-SSM7 and Pelagibacter phage HTVC019P, HTVC011P, and SAR116 phage HMO-2011 were also abundant (Kang et al., 2013; Zhao et al., 2013). From the perspective of relative abundance, Shewanella bacteriophages are generally not abundant. The relative abundance of vB_SInP-X14 was not high, mainly detected in the MES and ARC, and not detected in BATHY and ANT. Vibrio_phage_1.083.O was the most abundant Shewanella phage, and mainly detected from the MES, BATHY, and ARC, and followed by SppYZU01, SFCi1, 1/44, and vB_SInP-X14. The distribution patterns of SppYZU01 and vB_SInP-X14 were similar. In addition, some Shewanella phages were not detected in any VEZs of the GOV2.0 dataset, including Shewanella phage SppYZU05, Shewanella_phage_Spp001, or Shewanella_phage_S0112 (Figure 6). Although the relative abundances of Shewanella phages are not high in the ocean, considering the ecological significance and sedimental preference of *Shewanella* and their ability in the dissimilatory metal reduction and the heavy metal treatment (Yang et al., 2020), Shewanella phages should still play significant roles in the ocean through the mediation of their abundance and community structure, especially in the sediments and metal pollution environments.

## Conclusion

Viruses play an important role in marine ecosystems by affecting their host’s physiology and ecology. Culturing novel viruses infecting major bacterial populations, such as *Shewanella*, is likely to provide important references for the virus–host linkage of viral sequences from metagenomic data (Lemaire et al., 2020). In this study, phage vB_SInP-X14, the first podovirus infecting *Shewanella*, was described in the aspect of morphology, growth, genomic, and phylogenetic characteristics. Phage vB_SInP-X14 is a lytic virus with a short reproduction time and three lytic-related ORFs, representing a new viral genus of podovirus, namely, *Bocovirus*. This study contributes to our knowledge of the little-known Shewanella phages, improves our understanding of the genomic characteristics, genetic diversity, and ecological distribution of phages in the sea, and contributes to the prediction of the viral sequences from the metagenomic database. Considering the significant roles and ability of *Shewanella* in the dissimilatory metal reduction and the heavy metal treatment, the isolation, abundance, and ecological roles of Shewanella phages should be paid special attention in the future, especially in the sediments and metal pollution environments (Fredrickson et al., 2008; Lemaire et al., 2020; Cao et al., 2021).

## Data Availability Statement

The datasets presented in this study can be found in online repositories. The names of the repository/repositories and accession number(s) can be found in the article/Supplementary Material.

## Author Contributions

YLa and MW: conceptualization, revision, project administration, supervision and funding acquisition. YD, KZ, and XZ: methodology, formal analysis, writing, and original draft preparation. YLu and XL: phage isolation, software, validation, and visualization. HS, YS, WM, LW, and AM: review and editing. All authors contributed to the article and approved the submitted version.

## Conflict of Interest

The authors declare that the research was conducted in the absence of any commercial or financial relationships that could be construed as a potential conflict of interest.

## Publisher’s Note

All claims expressed in this article are solely those of the authors and do not necessarily represent those of their affiliated organizations, or those of the publisher, the editors and the reviewers. Any product that may be evaluated in this article, or claim that may be made by its manufacturer, is not guaranteed or endorsed by the publisher.

## Footnotes

1
https://genskew.csb.univie.ac.at/webskew

2
https://www.genome.jp/viptree
