# Supplementary material for: Characterization and Genomic Analysis of the First Podophage Infecting Shewanella, Representing a Novel Viral Cluster
Source: Front Microbiol. 2022 Apr 1;13:853973. doi: 10.3389/fmicb.2022.853973 (PMC9011153; doi:10.3389/fmicb.2022.853973)
Supplement: Supplementary file 1 [file Data_Sheet_1.docx]

Supplementary Material

# Supplementary Figures and Tables

- 1. **Supplementary Figures**


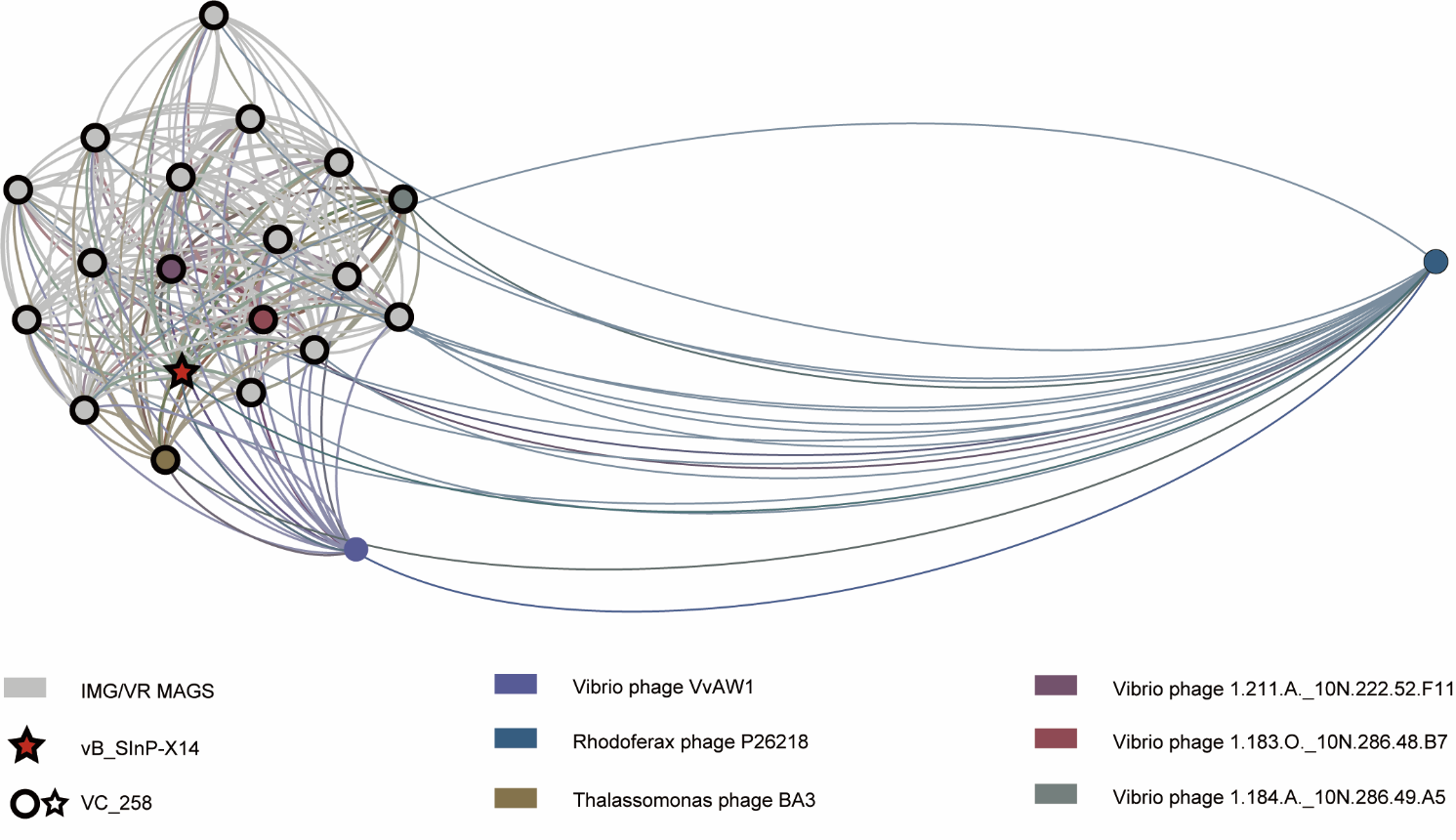


**Supplementary Figure 1.** Gene-content-based viral network of Shewanella phage vB_SInP-X14, the 65,899*Caudovirales* virus from NCBI database, and related UViGs from IMG/VR dataset. The results only showed that it was similar to vB_SInP-X14 has directly linked phages.


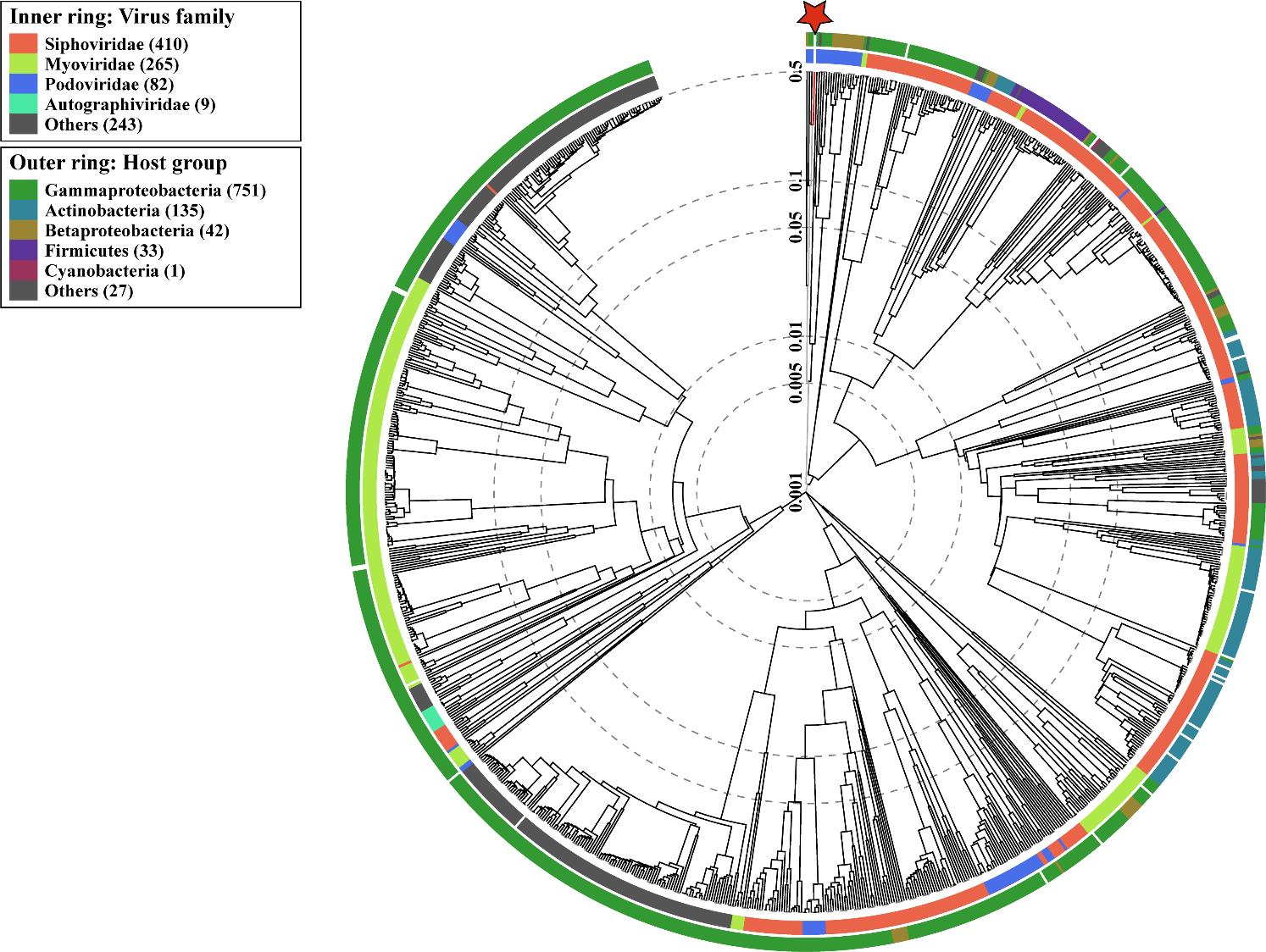


**Supplementary Figure 2.** Phylogenetic tree of all isolated Shewanella phages by VipTree. The red star is Shewanella phage vB_SInP-X14


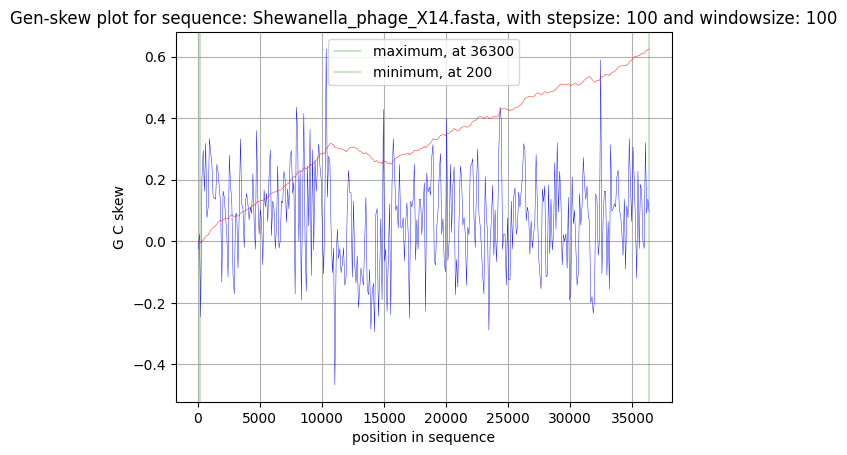


**Supplementary Figure 3.** GC-associated genome analysis. In the GC skew, the low region in GC is indicated by the bar. In the cumulative GC skew, the origin of replication (minimum, at 200) and the termination of DNA replication (maximum, at 36,300) are indicated.

## Supplementary Tables

TABLE S1| Genomic annotation of Shewanella phage vB_SInP-X14 and conserved domains detected based on homology to Pfam, PDB, COG, NCBI.

|  |  |  |  |  |  |
| --- | --- | --- | --- | --- | --- |
| ORF | Start | Stop | Annotation | CDs/Accession | Evalue |
| 1+ | 1 | 8223 | DNA methylase subunit | COG0286 | 3.9E-10 |
| 3- | 11376 | 10720 | Bacteriophage Lambda NinG protein | PF05766.13 | 1.00E-31 |
| 5- | 12358 | 11816 | HNH endonuclease | PF13392.7 | 1.10E-13 |
| 7- | 13281 | 12754 | DNA N-6-adenine-methyltransferase | PF05869.14 | 7.10E-26 |
| 8- | 13987 | 13271 | DNA-methyltransferase | KOG0919 | 4.40E-20 |
| 9- | 14574 | 13984 | Replication protein P | PF06992.14 | 4.10E-19 |
| 11- | 15588 | 15385 | Helix-turn-helix | PF13443 | 1.2E-08 |
| 14+ | 16348 | 17103 | DNA single-strand annealing protein | PF04404.13 | 4.3E-24 |
| 15+ | 17105 | 19069 | DNA single-strand annealing protein | PF04404.15 | 4.3E-24 |
| 16+ | 19123 | 19683 | Exonuclease (DNA polymerase III) | COG2176 | 3E-22 |
| 17+ | 19692 | 20423 | DNA-binding domain protein | YP_009275959.1 | 2E-41 |
| 18+ | 20398 | 20655 | Adhesin biosynthesis transcription regulatory protein | PF03333.14 | 1.9E-20 |
| 22+ | 21443 | 21997 | N-acetylmuramoyl-L-alanine amidase | PF01520.21 | 1.6E-10 |
| 24+ | 22151 | 22660 | Holin of 3TMs | PF11351.11 | 7.6E-12 |
| 25+ | 22928 | 23134 | Holin family protein | PF16085.8 | 1.3E-09 |
| 27+ | 23628 | 24263 | Terminase small subunit | PF03592.19 | 5.5E-25 |
| 28+ | 24442 | 26076 | Terminase large subunit | 5OE8_B (PDB) | 3E-32 |
| 31+ | 26375 | 28525 | P22-like portal protein | PF16510.8 | 1.8E-56 |
| 32+ | 28617 | 29534 | Bacteriophage scaffolding protein | PF09306.13 | 1.6E-28 |
| 33+ | 29555 | 30805 | P22-like coat protein | PF11651.11 | 8.2E-61 |
| 35+ | 31405 | 32169 | P22 tail accessory factor | PF11650.11 | 3.9E-08 |
| 36+ | 32171 | 33544 | Phage stabilisation protein | PF11134.11 | 3.8E-49 |
| 38+ | 34235 | 34795 | Unknown function structural gene | 3HQX(PDB) | 1.8E-11 |
| 39+ | 34786 | 35475 | Unknown function structural gene | 3HQX(PDB) | 1.8E-11 |

TABLE S2| Information of 14 UViG sequences from IMG/VR database.

| UViG | Ecosystem | MIUViG quality | Taxonomic classification | Taxonomic classification method |
| --- | --- | --- | --- | --- |
| DTR_657540 | Marine | Reference | Podoviridae | Viral RefSeq genes |
| IMGVR_UViG_3300005588_000026 | Marine(sedient) | High-quality | Podoviridae | Viral RefSeq genes |
| IMGVR_UViG_3300005589_000019 | Marine(sedient) | High-quality | Podoviridae | Viral RefSeq genes |
| IMGVR_UViG_3300006467_000468 | Marine | High-quality | Podoviridae | Viral RefSeq genes |
| IMGVR_UViG_3300007540_000005 | Marine | High-quality | Podoviridae | Viral RefSeq genes |
| MGVR_UViG_3300007538_000013 | Marine | High-quality | Podoviridae | Viral RefSeq genes |
| IMGVR_UViG_3300009193_000004 | Marine | High-quality | Podoviridae | Viral RefSeq genes |
| IMGVR_UViG_3300017991_000380 | Hypersaline;Aquatic(sediment) | Genome fragment(67.14%) | Podoviridae | Viral RefSeq genes |
| IMGVR_UViG_3300022200_000014 | Marine | High-quality | Podoviridae | Viral RefSeq genes |
| IMGVR_UViG_3300025137_000488 | Marine | Genome fragment(49.42%) | Podoviridae | Viral RefSeq genes |
| IMGVR_UViG_3300025543_000004 | Marine | High-quality | Podoviridae | Viral RefSeq genes |
| IMGVR_UViG_3300025873_000001 | Marine | High-quality | Podoviridae | Viral RefSeq genes |
| IMGVR_UViG_3300027980_000026 | Marine(sedient) | High-quality | Podoviridae | Viral RefSeq genes |
| Station168_DCM_ALL_assembly_NODE_2128_length_36930_cov_593.410278 | Marine | High-quality | Podoviridae | Viral RefSeq genes |
